# Fumaric Acid Esters Can Block Pro-Inflammatory Actions of Human CRP and Ameliorate Metabolic Disturbances in Transgenic Spontaneously Hypertensive Rats

**DOI:** 10.1371/journal.pone.0101906

**Published:** 2014-07-10

**Authors:** Jan Šilhavý, Václav Zídek, Petr Mlejnek, Vladimír Landa, Miroslava Šimáková, Hynek Strnad, Olena Oliyarnyk, Vojtěch Škop, Ludmila Kazdová, Theodore Kurtz, Michal Pravenec

**Affiliations:** 1 Institute of Physiology, Academy of Sciences of the Czech Republic, Prague, Czech Republic; 2 Institute of Molecular Genetics, Academy of Sciences of the Czech Republic, Prague, Czech Republic; 3 Center for Experimental Medicine, Institute for Clinical and Experimental Medicine, Prague, Czech Republic; 4 University of California San Francisco, San Francisco, California, United States of America; Institute of Medical Research A Lanari-IDIM, University of Buenos Aires-National Council of Scientific and Technological Research (CONICET), Argentina

## Abstract

Inflammation and oxidative stress have been implicated in the pathogenesis of metabolic disturbances. Esters of fumaric acid, mainly dimethyl fumarate, exhibit immunomodulatory, anti-inflammatory, and anti-oxidative effects. In the current study, we tested the hypothesis that fumaric acid ester (FAE) treatment of an animal model of inflammation and metabolic syndrome, the spontaneously hypertensive rat transgenically expressing human C-reactive protein (SHR-CRP), will ameliorate inflammation, oxidative stress, and metabolic disturbances. We studied the effects of FAE treatment by administering Fumaderm, 10 mg/kg body weight for 4 weeks, to male SHR-CRP. Untreated male SHR-CRP rats were used as controls. All rats were fed a high sucrose diet. Compared to untreated controls, rats treated with FAE showed significantly lower levels of endogenous CRP but not transgenic human CRP, and amelioration of inflammation (reduced levels of serum IL6 and TNFα) and oxidative stress (reduced levels of lipoperoxidation products in liver, heart, kidney, and plasma). FAE treatment was also associated with lower visceral fat weight and less ectopic fat accumulation in liver and muscle, greater levels of lipolysis, and greater incorporation of glucose into adipose tissue lipids. Analysis of gene expression profiles in the liver with Affymetrix arrays revealed that FAE treatment was associated with differential expression of genes in pathways that involve the regulation of inflammation and oxidative stress. These findings suggest potentially important anti-inflammatory, anti-oxidative, and metabolic effects of FAE in a model of inflammation and metabolic disturbances induced by human CRP.

## Introduction

Fumaderm is a preparation of fumaric acid esters (FAE), mainly dimethyl fumarate (DMF) and monomethyl fumarate (MMF) salts approved for treatment of psoriasis vulgaris in Germany and some neighboring countries [1]. Owing to its immunomodulatory and anti-inflammatory effects, DMF was recently approved by the US Food and Drug Administration as a first-line therapy for adults with relapsing forms of multiple sclerosis. In addition, DMF has been explored for the treatment of other diseases including sarcoidosis, necrobiosis lipoidica or granuloma annulare and has also been studied in a variety of animal models including disorders such as cancer, malaria, and Huntington disease [1].

Inflammation and oxidative stress have been implicated in the pathogenesis of obesity, metabolic disturbances, diabetes, and cardiovascular disease [2]. Recently, we derived a new strain of “humanized” spontaneously hypertensive rats (SHR-CRP) in which transgenic expression of human C-reactive protein (CRP) in liver induces inflammation, oxidative stress, several features of metabolic syndrome, and target organ damage [3]. In the current study, we explored whether FAE can exert anti-inflammatory and anti-oxidative actions associated with metabolic effects in this animal model.

## Results

### Fumaric Acid Esters Ameliorated Inflammation in Transgenic SHR-CRP Rats

**Figure 1:**
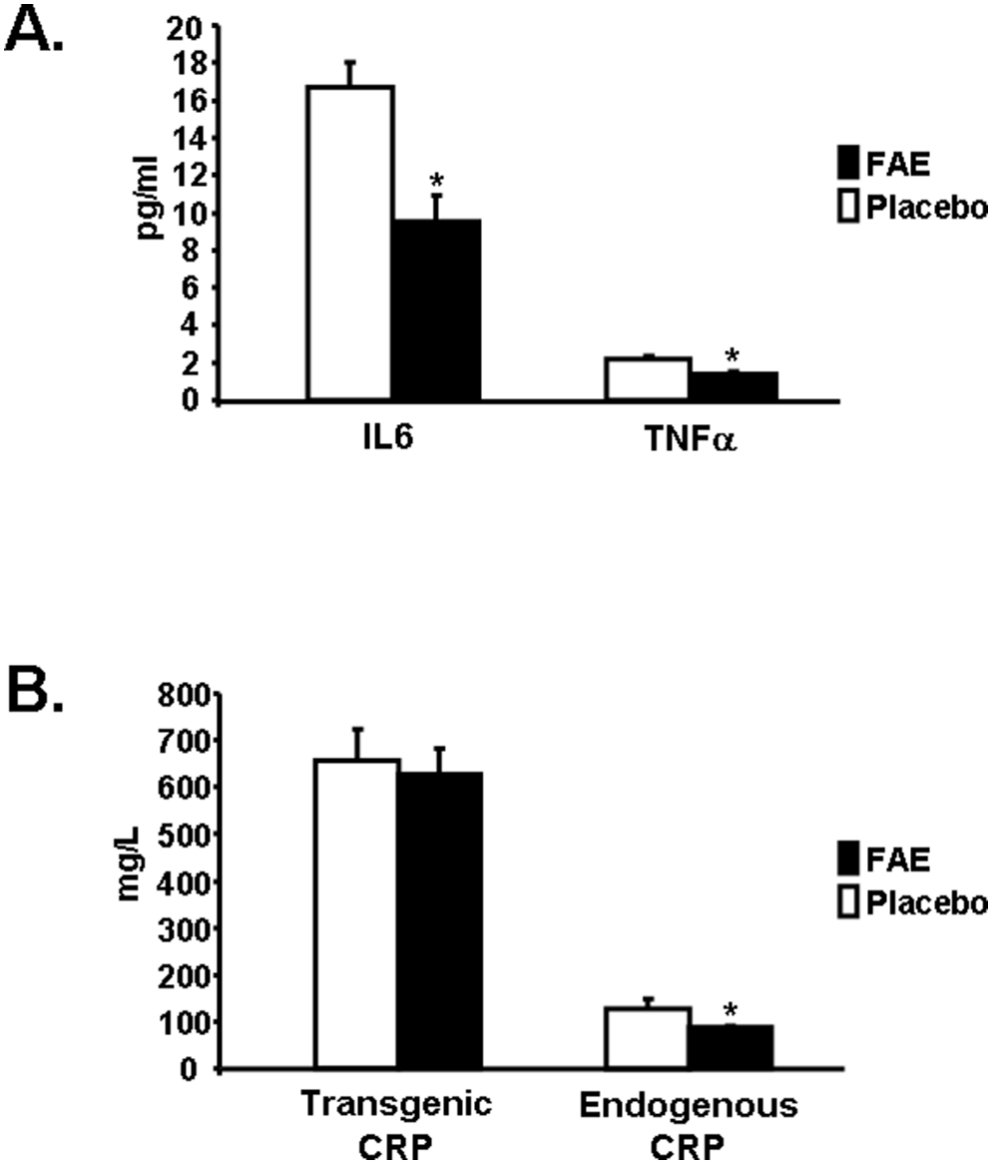
Serum levels of inflammatory markers. A. Serum levels of IL6 and TNFα in fumaric acid ester (FAE) treated SHR-CRP transgenic rats (solid bars) (N = 6) were significantly reduced when compared to untreated controls (N = 7). B. Serum levels of transgenic human CRP were similar in FAE treated rats (solid bars) when compared to untreated rats (open bar). On the other hand, rat endogenous CRP was significantly reduced in FAE treated rats (P<0.05).

Rats treated with fumaric acid esters (FAE) exhibited reduced inflammation as suggested by lower levels of inflammatory markers IL6 and TNFα (Figure 1A). Levels of transgenic CRP were similar in treated versus control rats (Figure 1B) while levels of endogenous rat CRP were significantly lower in FAE treated rats than in control rats (Figure 1B). Next we assessed the effects of FAE treatment on endogenous rat CRP in the nontransgenic SHR strain. In the nontransgenic SHR strain treated with FAE, the serum level of endogenous rat CRP tended to be greater than in the untreated nontransgenic SHR strain (260±14 vs. 227±20 mg/L, respectively, P = 0.14). Thus, FAE treatment per se does not lower endogenous rat CRP. In contrast, in the SHR-CRP transgenic strain treated with FAE, the serum level of endogenous rat CRP was significantly lower than in the untreated SHR-CRP transgenic strain (87±5 vs. 129±19 mg/L, respectively, P<0.05). Note that in the SHR-CRP transgenic strain, the serum levels of endogenous rat CRP are lower than those in the nontransgenic SHR strain regardless of drug treatment. It is possible that the generally lower level of endogenous rat CRP in the transgenic strain is secondary to overexpression of the human CRP transgene. Two way ANOVA thus showed significant strain effects on endogenous CRP levels (P<0.0001) while the overall effects of FAE treatment on endogenous rat CRP levels were not significant (P = 0.76).

### Effects of Fumaric Acid Esters on Oxidative Stress Related Parameters

In liver and renal cortex, the activity of the antioxidative enzyme SOD (superoxide dismutase) was significantly greater in FAE treated rats compared to controls (Table 1). In liver and heart tissue, the activities of GSH-dependent enzymes, GSH-Px (glutathione peroxidase) and GST (glutathione transferase), were also greater in FAE treated rats than in controls. The activity of the GSH-regenerating enzyme GR (glutathione reductase) was elevated in plasma of the FAE treated rats but the concentration of GSH (reduced glutathione) in tissues remained unchanged. The activity of catalase was greater in liver, renal cortex, and plasma in treated rats compared to controls. The greater levels of antioxidative enzyme activity were associated with amelioration of oxidative stress as the levels of lipoperoxidation products measured by TBARS (thiobarbituric acid reactive substances) were lower in plasma, liver, myocardium, and renal cortex of treated rats versus controls (Table 1).

**Table 1:** Parameters of oxidative stress associated with fumaric acid esters (FAE) treatment.

Tissue	SHR-CRP control	SHR-CRP treated with FAE
**Superoxide dismutase**		
Plasma (U/ml)	1.79±0.16	1.79±0.14
Liver (U/mg protein)	0.129±0.010	0.165±0.009*
Myocardium (U/mg protein)	0.047±0.006	0.050±0.003
Renal cortex (U/mg protein)	0.030±0.003	0.068±0.005**
**Glutathione peroxidase**		
Plasma (µmol NADPH min/ml)	186±11	163±6
Liver (µmol NADPH min mg protein)	208±17	292±18**
Myocardium (µmol NADPH/min/mg protein)	82±2	103±4**
Renal cortex (µmol NADPH min/mg protein)	129±6	178±6**
**Glutathione transferase**		
Plasma (nmol CDNB/min/ml)	4.42±0.40	5.00±0.28
Liver (nmol CDNB/min/mg protein)	182±19	239±7*
Myocardium (nmol CDNB/min/mg protein)	25±2	32±1**
Renal cortex (nmol CDNB/min/mg protein)	52±3	53±3
**Glutathione reductase**		
Plasma (µmol NADPH/min/ml)	98±6	134±9*
Liver (µmol NADPH/min/mg protein)	133±15	110±12
Myocardium (µmol NADPH/min/mg protein)	45±4	44±4
Renal cortex (µmol NADPH/min/mg protein)	42±3	46±3
**Reduced glutathione**		
Plasma (µmol/ml)	3.4±0.2	3.3±0.1
Liver (mmol/mg protein)	34.3±2.1	37.7±3.5
Myocardium (mmol/mg protein)	18.9±0.9	17.9±0.9
Renal cortex (mmol/mg protein)	14.3±0.9	15.4±1.3
**Catalase**		
Plasma (µmol H_2_O_2_/min/ml)	1166±64	1442±79*
Liver (µmol H_2_O_2_/min/mg protein)	1136±25	1346±30**
Myocardium (µmol H_2_O_2_/min/mg protein)	617±44	600±31
Renal cortex (µmol H_2_O_2_/min/mg protein)	441±19	534±32*
**TBARS**		
Plasma (nmol/ml)	1.861±0.228	1.221±0.105*
Liver (nmol/mg protein)	1.701±0.110	1.273±0.58**
Myocardium (nmol/mg protein)	0.900±0.039	0.777±0.021*
Renal cortex (nmol/mg protein)	0.962±0.030	0.685±0.048**

** and * denote p<0.001 and p<0.05, respectively. Abbreviations: CDNB, 1-Chloro-2,4-dinitrobenzene; TBARS, thiobarbituric acid reactive substances.

### Metabolic and Hemodynamic Effects of Fumaric Acid Esters

**Figure 2:**
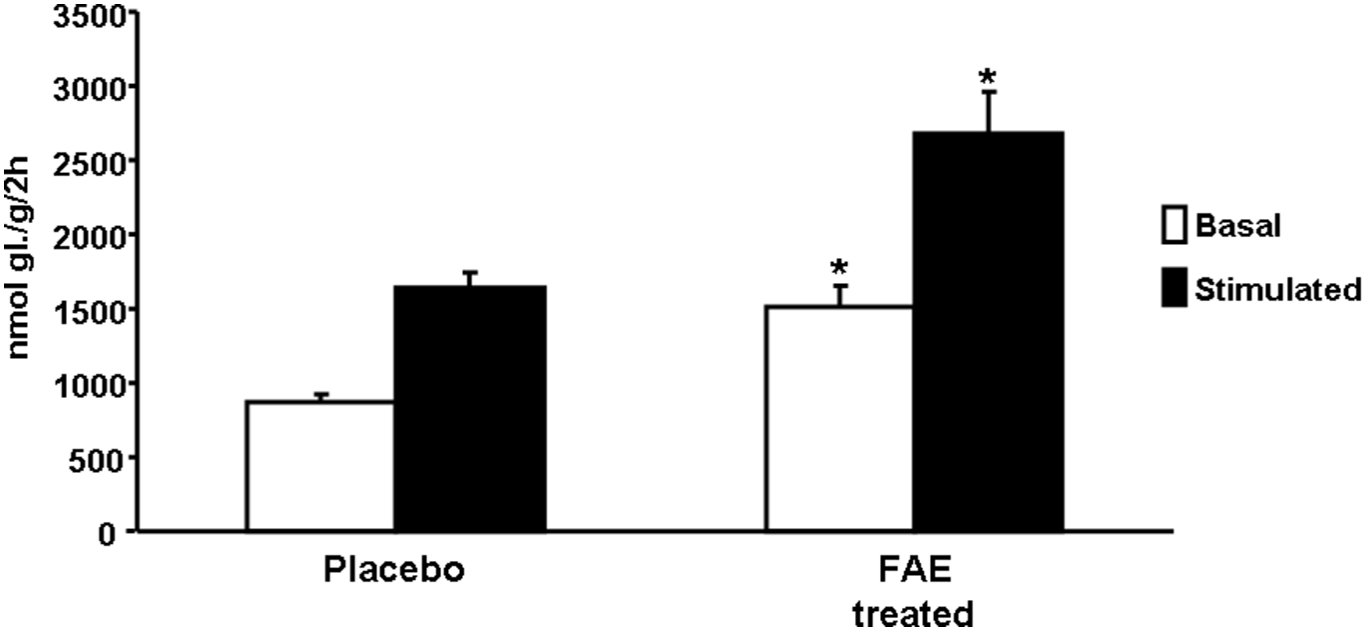
Basal and insulin stimulated lipogenesis in SHR-CRP transgenic rats treated with fumaric acid esters (FAE) (N = 6) or placebo (N = 7). FAE treated SHR-CRP transgenic rats showed significantly greater levels of both basal (open bars) and insulin stimulated (solid bars) incorporation of radioactively labeled glucose into adipose tissue lipids when compared to untreated rats. *denotes significant difference compared to untreated controls, P<0.01.

**Figure 3:**
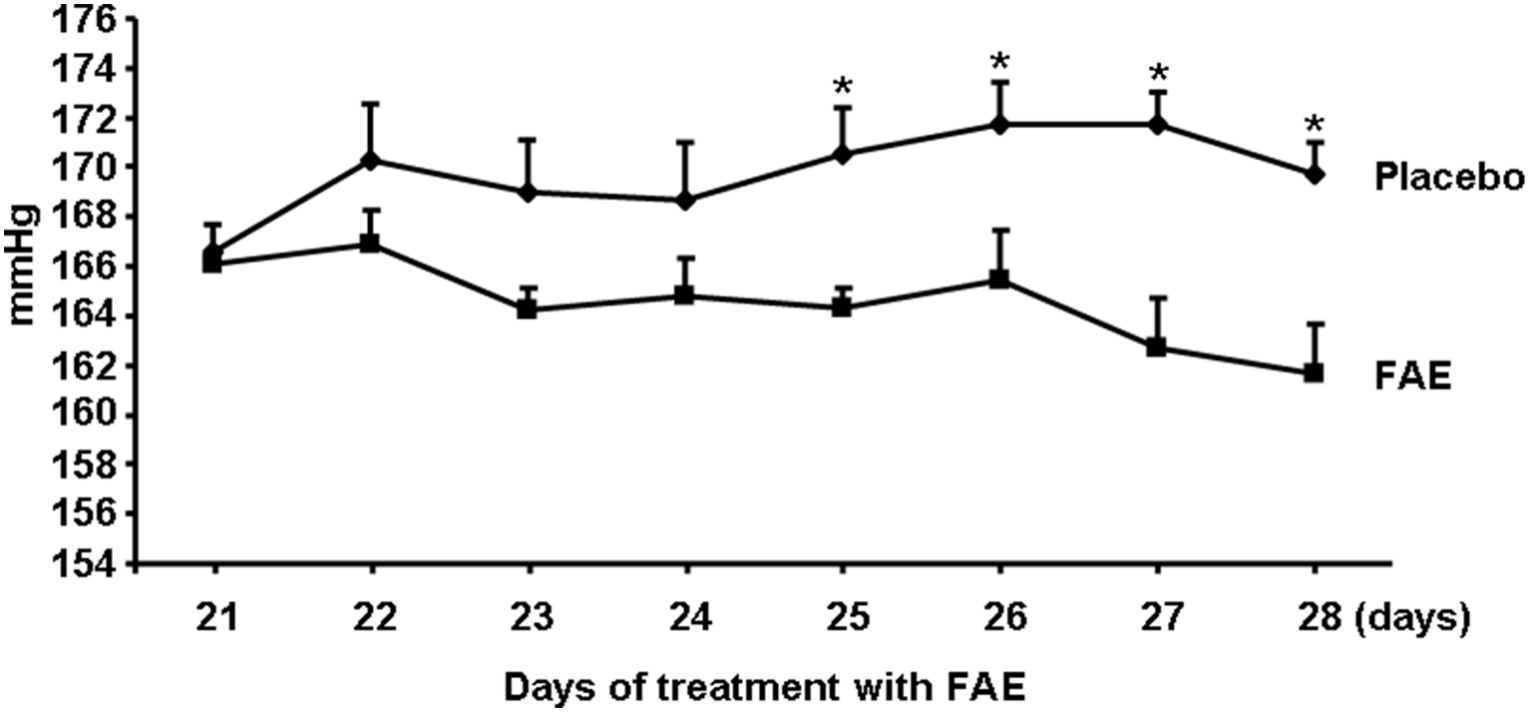
Systolic blood pressures. The daily 24-hour average systolic blood pressures measured by radiotelemetry in conscious, unrestrained transgenic SHR-CRP rats treated with fumaric acid esters (FAE) (N = 8) were significantly greater than in untreated transgenic SHR-CRP controls (N = 8) (*denotes P<0.01).

As shown in Table 2, FAE treatment appeared to be associated with reduced adiposity as reflected by lower weight of epididymal fat, and reduced ectopic fat accumulation in liver and skeletal muscle. FAE treatment was also associated with significantly increased adrenaline stimulated lipolysis and higher levels of serum NEFA and triglycerides. SHR-CRP treated with FAE showed significantly greater levels of both basal and insulin stimulated incorporation of glucose into adipose tissue lipids when compared to untreated controls (Figure 2). There were no significant differences between FAE treated and control rats in insulin stimulated incorporation of glucose into muscle glycogen (Table 2). There were no significant differences in plasma glucose and insulin between treated and control rats. On the other hand, FAE treated rats had significantly higher levels of adiponectin when compared to untreated controls (Table 2). No significant differences were observed in food consumption between experimental groups (data not shown). Systolic blood pressures measured by telemetry were reduced in rats after treatment with FAE for 4 weeks when compared to untreated controls (Figure 3) but there were no significant differences in distolic blood pressures (data not shown).

**Table 2:** Metabolic parameters in SHR-CRP transgenic rats treated with fumaric acid esters (FAE) or placebo.

Trait	SHR-CRP placebo	SHR-CRP treated with FAE
Body weight (g)	407±7	405±12
Relative liver weight (g/100 g BW)	3.89±0.12	3.88±0.12
Relative epididymal fat weight (g/100 g BW)	0.94±0.02	0.73±0.05**
Plasma trigylcerides (mmol/L)	1.08±0.13	1.42±0.06*
Plasma NEFA (mmol/L)	0.35±0.03	0.59±0.05**
Plasma glucose (mmol/L)	8.6±0.4	8.4±0.3
Plasma insulin (nmol/L)	0.73±0.11	0.70±0.06
Plasma adiponectin (ng/mL)	8.2±0.5	10.1±0.5*
Liver triglycerides (nmol/g)	25.7±4.1	14.2±1.2*
Heart triglycerides (nmol/g)	1.62±0.20	1.64±0.13
Muscle triglycerides (nmol/g)	3.10±0.17	2.41±0.25*
Basal lipolysis NEFA (µmol/g)	3.26±0.30	3.33±0.42
Adrenaline stimulated lipolysis NEFA (µmol/g)	5.91±0.90	9.27±1.04*
Basal glycogenesis (nmol gl./g/2 h)	70.8±11.9	54.7±6.8
Insulin stimulated glycogenesis (nmol gl./g/2 h)	231.4±16.8	247.9±10.8

** and * denote p<0.005 and p<0.05, respectively. Abbreviations: BW, body weight; NEFA, nonesterified fatty acids.

### Gene Expression Profiles

**Figure 4:**
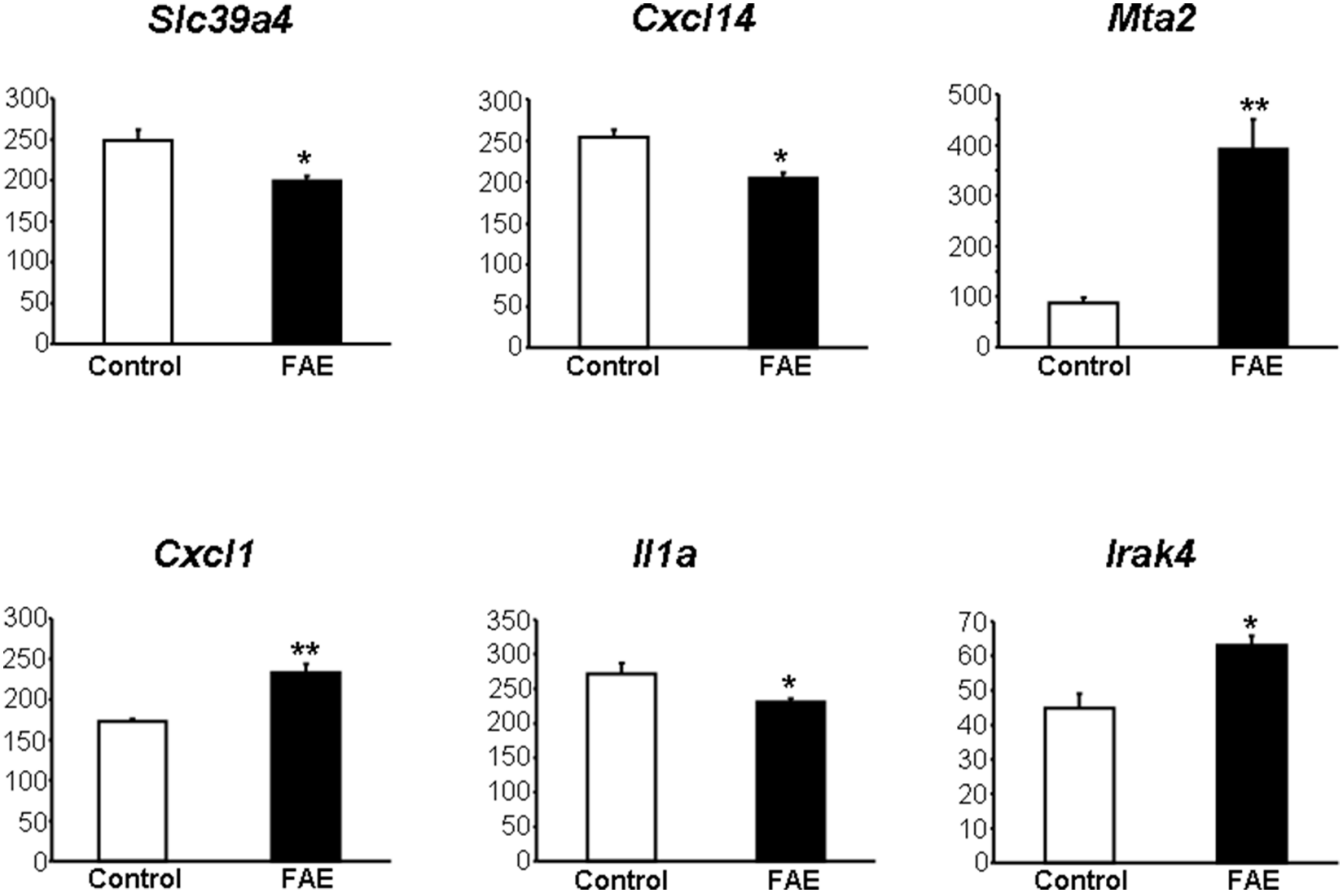
Validation of gene expression profiles obtained by Affymetrix transcriptional profiling by quantitative real time PCR for six transcripts in livers isolated from SHR-CRP rats treated with fumaric acid esters (FAE) (solid bars) versus untreated SHR-CRP controls (open bars). Expression of selected genes was normalized relative to the expression of the peptidylprolyl isomerase A (*Ppia*) gene, which served as an internal control.

Altogether, almost 1500 genes were differentially expressed at a nominal significance value of P<0.05, but after correction for multiple testing, these differences were not statistically significant. However, we were able to confirm directional differences in expression of selected genes by real time PCR analysis (Figure 4). Since monomethyl fumarate can activate niacin receptor (coded by *Hcar2* gene), we also tested hepatic expression of *Hcar2* gene and found that it is downregulated in FAE treated rats when compared to untreated controls (normalized expression 9.3±0.6 vs. 13.8±0.7, P = 0.003). The GSEA and SPIA based screening of the KEGG pathway database identified significantly lower or higher expression of genes from KEGG pathways in FAE treated SHR-CRP rats versus SHR-CRP controls (Table 3). These pathways include genes related to immuno-modulatory and inflammatory pathways that show reduced expression in FAE treated rats compared untreated controls. Most of genes with lower expression from GSEA KEGG pathways play important roles in Jak-Stat and chemokine signaling (Table 3) and some of differentially expressed genes from the Leishmaniasis and Toxoplasmosis pathways belong to additional pro-inflammatory Toll-like receptor signaling pathway (*Irak4*, *Mapk14*, *Stat1*, *Cd40*, *Pik3r3*, *Pik3cb*, *Akt3*, *Map2k6*, *Cxcl9*, *Tlr4*, *Traf6*). Suppression of these pathways in FAE treated rats was associated with reduced inflammation. In addition, GSEA and SPIA identified increased expression of some genes from terpenoid backbone biosynthesis, steroid biosynthesis, and glutathione metabolism KEGG pathways, as well as from the mineral absorption pathway (*Mt1a*, *Mt2a*, *Hmox1*) that play important role in lipid metabolism and in protecting cells against oxidative stress (Table 3).

**Table 3:** KEGG pathways determined by GSEA and SPIA analysis.

GSEA on KEGG pathways(downregulated)	FDR (GSEA)	Deregulated genes (P<0.05)
Leishmaniasis	0.0025	*Irak4*, *RT1-Ba*, *Fcgr3a*, *RT1-Dma*, *Il1a*, *Jak2*, *RT1-DMb*, *Cyba*, *Mapk14*, *Prkcb*, *Stat1*, *Itga*, *Tlr4*, *Traf6*
Toxoplasmosis	0.0033	*Pla2g2d, Irak4, Hspa1b, RT1-Ba, Ldlr, Stat3, RT1-Dma, Jak2, Il10rb, RT1-DMb, Cd40, Ciita, Pik3r3, Mapk14, Hspa2, Stat1, Pik3cb, Akt3, Map2k6, Il10ra, Tlr4, Traf6*
Jak-STAT signaling	0.0147	*Stat5b, Stat3, Il6r, Jak3, Il15, Il4a, Jak2, Osmr, Il10rb, Lepr, Pik3r3, Stat4, Stat1, Pik3cb, Akt3, Cntfr, Csf3r, Ctf1, Il10ra*
Protein export	0.0147	*Sec63, Srp72, Srp54, Srpr, Hspa5*
Spliceosome	0.0147	*Naa38, Tra2a, Hspa1b, Tra2b, Srsf7, Srsf6, Srsf9, Hspa2, Smndc1, Lsm5, Snrpb2, Prpf38b, Tra2a, Srsf10, Rbmx, Plrg1, Sart1*
Antigen processing and presentation	0.0147	*Hspa1b, RT1-Ba, RT1-Dma, RT1-DMb, RT1-N2, Ciita, Hspa2, RT1-CE3, Psme1, RT1-M6-2, Hspa5, Tap1*
Chemokine signaling	0.0218	*Cxcl12, Stat5b, Stat3, Jak3, Jak2, Foxo3, Fgr, Pik3r3, Prkcz, Vav1, Prkcb, Stat1, Cxcl9, Pik3cb, Gng13, Akt3, Cxcl14, Cxcr5, Cxcl1, Prex1, Gngt1, Ccl24*
SNARE interactions in vesicular transport	0.0282	*Stx3, Snap29, Stx18, Stx2, Sec22b, Stx1b, Snap47, Bet1, Stx7,*
Cytosolic DNA sensing	0.0455	*Irf7, Il18, Zbp1, Pol3gl, Il33, Ripk3*
**GSEA on KEGG** **pathways (upregulated)**	**FDR (GSEA)**	**Deregulated genes (P<0.05)**
Terpenoid backbone biosynthesis	0.000038	*Hmgcr, Acat1, Fdps, Pmvk, Acat3, Idi1, Mvd, Hmgcs1*
Steroid biosynthesis	0.00029	*Sc5dl, Soat1, Dhcr7, Lss, Cyp51, Hsd17b7, Msmo1, Sqle, Dhcr24, Soat2*
Glutathione metabolism	0.037	*Gss, Gclm, Gstp1, Gclc, Oplah, Mgst2, Gpx2, Ggt5, Gpx4, Idh2, Gstm3*
**SPIA on KEGG pathway** **(deregulated)**	**FWER (SPIA)**	**Deregulated genes (P<0.05)**
Mineral absorption	0.042	*Mti1, Mt2a, Hmox1, Slc30a1, Atp2b1, Slc39a4, Slc34a2, Cybrd1, Slc11a1*

KEGG pathways down- and upregulated in fumaric acid esters (FAE) treated SHR-CRP versus SHR-CRP controls; FWER – Family Wise Error Rate.

## Discussion

Fumaric acid esters (FAE) such as dimethyl fumarate (DMF) have potent anti-oxidative and anti-inflammatory effects [1], [4]. Inflammation and oxidative stress play important roles in the pathogenesis of obesity, diabetes, and related metabolic and cardiovascular disorders [2], [5]. There is also evidence indicating that increased levels of CRP may not only reflect the presence of inflammation, but also may promote inflammation and the risk for features of the metabolic syndrome and diabetes [3], [6], [7]. Therefore, in the current study in an animal model with inflammatory and metabolic disturbances induced by transgenic expression of human CRP, we tested the anti-inflammatory, anti-oxidative, and metabolic effects of Fumaderm, a preparation of fumaric acid esters containing DMF. We found that in the SHR-CRP rat model in which inflammation is known to be caused by increased expression of human CRP [3], FAE treatment was associated with significant anti-inflammatory effects despite the fact that treatment did not reduce circulating levels of transgenic human CRP. These findings are consistent with the possibility that FAE is protecting against the pro-inflammatory effects of human CRP. FAE treatment was associated with lower serum levels of endogenous rat CRP which likely reflects the anti-inflammatory effects of the drug. Given that endogenous rat CRP does not effectively fix complement and given that FAE treatment did not reduce endogenous rat CRP in nontransgenic SHR, it does not seem likely that the anti-inflammatory effects of FAE are being mediated by FAE induced decreases in endogenous rat CRP. Anti-inflammatory effects of FAE treatment appeared to be associated with significantly lower levels of oxidative stress as indicated by significantly lower levels of lipoperoxidation products in tissues. Amelioration of inflammation and oxidative stress in FAE treated rats was associated with less adiposity and ectopic fat accumulation, greater levels of lipolysis, and greater incorporation of glucose into adipose tissue lipids.

To search for molecular mechanisms associated with anti-inflammatory, anti-oxidative, and metabolic effects of FAE, we analyzed gene expression profiles in livers isolated from treated rats versus untreated controls. We focussed on liver because this is the main tissue site of expression of the human CRP transgene. We observed that FAE treatment was associated with downregulated Jak-Stat signaling, Toll-like receptor signaling, chemokine signaling KEGG pathways and with upregulated terpenoid backbone biosynthesis, steroid biosynthesis, and glutathione metabolism pathways, as well as deregulated mineral absorption pathway.

The Jak-Stat signaling pathway is the main intracellular cascade initiated in response to binding of cytokines to their receptors. Jak phosphorylation of Stats is followed by their translocation to the nucleus where they can regulate the expression of specific target genes [8]. In addition, the JAK2/STAT3 pathway is involved in the early stage of 3T3-L1 adipocyte differention [9]. Recently, Kang et al. [10] demonstrated in 3T3-L1 preadipocytes that DMF may function as an inhibitor of STAT3 and thus DMF is a negative regulator of adipogenic differentiation. These findings are in agreement with reduced adiposity and ectopic fat accumulation in rats treated with FAE. The Toll-like receptor signaling pathway regulates innate immune responses to various exogenous as well as endogenous stimuli by inducing the expression of many factors including pro-inflammatory cytokines, type I interferons, chemokines, and other molecules [11]. Chemokines play important roles in regulating inflammation by guiding cells of both the innate immune system and the adaptive immune system [12]. The fact that we observed downregulation of these pathways in treated rats suggests possible molecular mechanisms by which FAE protects against pro-inflammatory effects of transgenic CRP.

FAE treatment was associated with upregulated terpenoid backbone biosynthesis, steroid biosynthesis, and glutathione metabolism pathways (Table 3). Glutathione (GSH) is a major antioxidant and FAE treatment was associated with higher expression of genes involved in GSH biosynthesis: *Gclc* and *Gclm* genes that code for the catalytic and modifier subunits, respectively, of GCL (γ-glutamylcysteine synthetase) which catalyzes the first, rate limiting step in GSH synthesis and *Gss* (glutathione synthetase) that catalyzes the second step in GSH synthesis. Mineral absorption was the only identified significant SPIA KEGG pathway which contains genes important for regulation of oxidative stress including upregulated metallothionein *Mt1a* and *Mt2a* and *Hmox1* (heme oxygenase 1) genes.

It has been reported that DMF exerts antioxidative effects via NFE2L2 (also known as NRF2) (Nuclear factor (erythroid-derived 2)-like 2) transcription factor [13]–[15]. Upon activation, NRF2 translocates to the nucleus and binds to the Antioxidant Response Element (ARE) in the upstream promoter region of many antioxidative genes including *Mt1a*, *Mt2a*, *Hmox1*, *Gclc*, *Gclm*, *Gss*, *Gstp1*, *Gpx2*, *Ggt5*, *Gpx4*, and *Gstm3*. Some of these genes showed differential expression in treated versus control rats (Table 3), however, we observed no significant changes in the expression of *Nfe2l2* gene after FAE treatment.

DMF is converted in the intestine to monomethyl fumarate (MMF) which is the major active pharmacological substance [16]. Recently, MMF was found to be a potent agonist of the niacin receptor (known as GPR109A, HCA_2_, *Hcar2* or *Niacr1*) [17]. In addition, treatment with both niacin and DMF is associated with similar adverse side effects such as skin flushing which is dependent on niacin receptor activation [18] and pleiotropic effects of niacin include amelioration of inflammation and oxidative stress. Thus it is conceivable that the anti-inflammatory and anti-oxidant effects of FAE observed in these studies might be mediated, at least in part, by the effects of the active metabolite MMF on the niacin receptor [19]. On the other hand, we found that SHR-CRP rats treated with FAE showed reduced expression of *Hcar2* gene when compared to untreated controls which suggests that FAE does not activate niacin receptor.

In conclusion, the current findings provide evidence for potentially important actions of FAE on adipose tissue biology together with anti-inflammatory and anti-oxidative effects in a model of inflammation and metabolic disturbances induced by human CRP. Although the exact mechanisms mediating such actions of FAE in this model remain to be determined, the current studies raise the possibility that corresponding effects might be observed with FAE treatment in humans with metabolic disturbances associated with increased levels of CRP.

## Materials and Methods

### Animals

Transgenic SHR (hereafter referred to as SHR-CRP) were derived by microinjections of ova with a previously described construct containing the cDNA for human CRP under control of the apoE promoter [20] with the objective of driving expression of the CRP transgene in liver where CRP is normally produced [3]. We studied 2 groups of 16 month old male transgenic rats: 1) experimental group (N = 6) fed a high sucrose (60%) diet containing Fumaderm (Biogen Idec, Inc.) at a concentration of 500 mg Fumaderm/kg diet to deliver an approximate dose of 10 mg/kg body weight/day for 4 weeks, and 2) age matched, untreated control group (N = 7) fed the same high sucrose diet without Fumaderm for 4 weeks. We used also age-matched nontransgenic SHR to assess the effects of Fumaderm on rat endogenous CRP: 1) experimental group (N = 7) was treated with Fumaderm as transgenic rats and was compared to untreated SHR controls (N = 7). Because hypertension begins to develop at a relatively young age, blood pressure studies were performed in separate groups of 3 month old male SHR-CRP transgenic rats: 1) experimental group fed a high sucrose diet containing 500 mg Fumaderm/kg diet (N = 8) and age-matched untreated controls (N = 8). A high sucrose diet was used in these studies based on previous work indicating that such diets facilitate the development of metabolic disturbances in SHR models [21]. After the 4 week period of treatment, the rats were studied as described below. All rats were housed in an air-conditioned animal facility. All experiments were performed in agreement with the Animal Protection Law of the Czech Republic and were approved by the Ethics Committee of the Institute of Physiology, Academy of Sciences of the Czech Republic, Prague.

### Food Consumption

We measured daily food intake in each group by subtracting the amount of food remaining in the cage from the measured amount of food provided each day. The average daily food intake for each group was then calculated by averaging all of the daily intake measurements obtained over the entire course of the study.

### Basal and Insulin Stimulated Glycogen Synthesis in Skeletal Muscle

For measurement of insulin stimulated incorporation of glucose into glycogen, diaphragmatic muscles were incubated for 2 hours in 95% O_2_+5% CO_2_ in Krebs-Ringer bicarbonate buffer, pH 7.4, containing 0.1 µCi/ml of ^14^C-U glucose, 5 mmol/L of unlabeled glucose, and 2.5 mg/ml of bovine serum albumin (Sigma, Fraction V, Czech Republic), with or without 250 µU/ml insulin. Glycogen was extracted, and insulin stimulated incorporation of glucose into glycogen was determined.

### Glucose Utilization in Isolated Epididymal Adipose Tissue

Glucose utilization in adipose tissue was determined *ex vivo* by measuring the incorporation of radioactive glucose into adipose tissue lipids. Distal parts of epididymal adipose tissue were rapidly dissected and incubated for 2 hours in Krebs-Ringer bicarbonate buffer with 5 mmol/L glucose, 0.1 µCi ^14^C-U-glucose/mL (UVVR, Prague, Czech Republic) and 2% bovine serum albumin, gaseous phase 95% O_2_ and 5% CO_2_ in the presence (250 µU/mL) or absence of insulin in incubation media. All incubations were performed at 37°C in sealed vials in a shaking water bath. Estimation of ^14^C-glucose incorporation into neutral lipids was performed as described previously [3]. Briefly, adipose tissue was removed from incubation medium, rinsed in saline, and immediately put into chloroform. The pieces of tissue were dissolved using a Teflon pestle homogenizer, methanol was added (chloroform: methanol 2∶1), and lipids were extracted at 4°C overnight. The remaining tissue was removed, KH_2_PO_4_ was added and a clear extract was taken for further analysis. An aliquot was evaporated, reconstituted in scintillation liquid, and the radioactivity measured by scintillation counting.

### Lipolysis in Isolated Epididymal Adipose Tissue

For measurement of basal and adrenaline stimulated lipolysis, the distal parts of epididymal adipose tissue were incubated in Krebs-Ringer phosphate buffer containing 3% bovine serum albumin (Sigma, Fraction V, Czech Republic) at 37°C, pH 7.4 with or without adrenaline (0.25 µg/ml). The tissue was incubated for 2 hours and the concentrations of NEFA in the medium were determined. Basal lipolysis was measured as NEFA levels after 2 hours incubation without adrenaline. Stimulated lipolysis was measured as NEFA levels in media after 2 hours incubation with adrenaline.

### Tissue Triglyceride Measurements

For determination of triglycerides in liver and soleus muscle, tissues were powdered under liquid N_2_ and extracted for 16 hours in chloroform: methanol, after which 2% KH_2_PO_4_ was added and the solution was centrifuged. The organic phase was removed and evaporated under N_2_. The resulting pellet was dissolved in isopropyl alcohol, and triglyceride content was determined by enzymatic assay (Erba-Lachema, Brno, Czech Republic).

### Biochemical Analyses

Rat serum CRP and human serum CRP were measured using ELISA kits (Alpha Diagnostics International, San Antonio, U.S.A.). Blood glucose levels were measured by the glucose oxidase assay (Erba-Lachema, Brno, Czech Republic) using tail vein blood drawn into 5% trichloracetic acid and promptly centrifuged. NEFA levels were determined using an acyl-CoA oxidase-based colorimetric kit (Roche Diagnostics GmbH, Mannheim, Germany). Serum triglyceride concentrations were measured by standard enzymatic methods (Erba-Lachema, Brno, Czech Republic). Serum insulin concentrations were determined using a rat insulin ELISA kit (Mercodia, Uppsala, Sweden). Serum IL6 and TNFα were measured by rat ELISA kits (BioSource International, Inc., Camarillo, U.S.A.).

### Parameters of Oxidative Stress

The activity of antioxidative enzymes and concentrations of lipoperoxidation products were measured as previously described [22]. The activity of superoxide dismutase (SOD) was analyzed using the reaction of blocking nitrotetrazolium blue reduction and nitroformazan formation. Catalase (CAT) activity measurement was based on the ability of H_2_O_2_ to produce with ammonium molybdate a color complex detected spectrophotometrically. The activity of seleno-dependent glutathione peroxidase (GSH-Px) was monitored by oxidation of gluthathione by Ellman reagent (0.01 M solution of 5,5′-dythiobis-2 nitrobenzoic acid). The level of reduced glutathione (GSH) was determined in the reaction of SH-groups using Ellman reagent. Glutathione reductase (GR) activity was measured by the decrease of absorbance at 340 nm using a millimolar extinction coefficient of 6220 M^−1^cm^−1^ for NADPH (using Sigma assay kit). Lipoperoxidation products were assessed by the levels of thiobarbituric acid reactive substances (TBARS) determined by assaying the reaction with thiobarbituric acid [22].

### Blood pressure measurement

Arterial blood pressures were measured continuously by radiotelemetry (Data Sciences International, St. Paul, U.S.A.) in paired experiments between conscious, unrestrained male rats. All rats were allowed to recover for at least 7 days after surgical implantation of radiotelemetry transducers before the start of blood pressure recordings. Pulsatile pressures were recorded in 5-second bursts every 10 minutes throughout the day and night, and 24-hour averages for systolic and diastolic arterial blood pressure were calculated for each rat. The results from each rat in the same group were then averaged to obtain the group means.

### Gene Expression Profiling

Total RNA was extracted from livers of SHR-CRP rats treated with Fumaderm or placebo (N = 3 per group). Quality and concentration of RNA were determined with a NanoDrop 2000 spectrophometer (Thermo Scientific). The RNA integrity was analyzed in an Agilent Bioanalyzer 2100. We included only samples judged to have an intact RNA profile. Affymetrix GeneChip Rat Gene 1.0 ST Array System was used for the microarray analysis following the standard protocol: 100 ng RNA was amplified with Ambion WT Expression Kit (Applied Biosystems), 5.5 µg single-stranded cDNA was labeled and fragmented with GeneChip WT Terminal Labeling and Hybridization (Affymetrix) and hybridized on the chip according to the manufacturer procedure. The analysis was performed in three replicates.

### Gene expression determined by real time PCR

Total RNA was extracted from liver using Trizol reagent (Invitrogen), and cDNA was prepared and analyzed by real-time PCR testing using QuantiTect SYBR Green reagents (Qiagen, Inc.) on an Opticon continuous fluorescence detector (MJ Research). Gene expression levels were normalized relative to the expression of peptidylprolyl isomerase A (*Ppia*) (cyclophilin) gene, which served as the internal control, with results being determined in triplicates. Primers used for validation of differentially expressed genes selected from significant pathways are given in Table S1.

### Statistical Analysis

The data are expressed as means ± SEM. Individual groups were compared by unpaired Student t-test. Normality of distribution was tested by Shapiro-Wilk method. We used two way ANOVA to search for strain (SHR-CRP transgenic versus SHR nontransgenic) and Fumaderm treatment effects on levels of rat endogenous CRP. The 24 hour mean values of systolic and diastolic blood pressures were analyzed by repeated measures ANOVA with grouping effect of treatment and repeated measurements in time. Statistical significance was defined as P<0.05.

Gene expression data were preprocessed in Partek Genomic Suit (Partek Incorporated). Analyses were performed using methods similar to those previously described [23]. Briefly, the transcription profiles were background corrected using the RMA method, probesets summarized by median polish, quantile normalized and variance stabilized using base-2 logarithmic transformation. Analysis of variance yielded transcripts differentially expressed between analyzed samples (within LIMMA) [24]. Storeýs q values [25] were used to select significant differentially expressed genes (q<0.05). The transcription data are MIAME compliant and deposited in the ArrayExpress database (ID #E-MTAB-2406).

All statistical analyses were performed in R and within Bioconductor [26]. Differentially expressed genes were selected for GSEA (Gene Set Enrichment Analysis) [27]. We performed GSEA on genes that mapped to KEGG pathways [28] and have defined GO terms [29] using the Fisher test and approach of Tian [30]. For the purpose of the GSEA, transcripts with P<0.05 were considered differentially expressed. To identify significantly perturbed pathways, we performed SPIA (Signaling Pathway Impact Analysis) [31] analysis on KEGG pathways: genes with P<0.05 were considered differencially transcribed. Where appropriate, the resulting statistical p-values were corrected for multiple testing by FDR method [32].

## Supporting Information

Table S1 **Primers used for RT PCR analysis.**
(XLS)
